# Supplementary material for: Discovery and application of insertion-deletion (INDEL) polymorphisms for QTL mapping of early life-history traits in Atlantic salmon
Source: BMC Genomics. 2010 Mar 8;11:156. doi: 10.1186/1471-2164-11-156 (PMC2838853; doi:10.1186/1471-2164-11-156)
Supplement: Additional file 2 — Information on developed 76 locus single-run INDEL panel in Atlantic salmon. Information on fluorescence labeling, primer concentrations, PCR pooling and links to alignments, INDEL motifs and GENESCAN (Burge and Karlin 1997) predictions of genes/exons are available in html format. [file 1471-2164-11-156-S2.ZIP › Additionalfile2/table11953.html]

Contig Alignment

```
Base	A B C D E F G H I J K L   
1	. G . . . . . . . . . .    
2	. G . . . . G . . . . .    
3	. T . . . . T . . . . .    
4	G G G G G . G . G . . G    
5	G G G G G . G . G . . G    
6	T T T T T . T . T . . T    
7	T T T T T . T . T . . T    
8	C C C C C . C . C . . C    
9	A A A A A . A . A . . A    
10	A A A A A . A . A . . A    
11	A A A A A . A . A . . A    
12	G G G G G . G . G . . G    
13	C C C C C . C . C . . C    
14	A A A A A . A . A . . A    
15	T T T T T . T . T . . T    
16	T T T T T . T . T . . T    
17	T T T T T . T . T . . T    
18	T T T T T . T . T . . T    
19	T T T T T . T . T . . T    
20	A A A A A . A . A . . A    
21	T T T T T . T . T . . T    
22	T T T T T . T . T . . T    
23	G G G G G . G . G . . G    
24	T T T T T . T . T . . T    
25	A A A A A . A . A . . A    
26	T T T T T . T . T . . T    
27	C C C C C . C . C . . C    
28	T T T T T . T . T . . T    
29	T T T T T . T . T . . T    
30	T T T T T . T . T . . T    
31	A A A A A . A . A . . A    
32	G G G G G . G . G A A G    
33	A A A A A . A . A A A A    
34	A A A A A . A . A A A A    
35	A A A A A . A . A A A A    
36	A A A A A . A . A A A A    
37	A A A A A . A . A A A A    
38	C C C C C . C . C C C C    
39	A A A A A . A . A A A A    
40	C C C C C . C . C C C C    
41	A A A A A . A . A A A A    
42	A A A A A . A . A A A A    
43	A A A A A . A . A A A A    
44	A A A A A . A . A A A A    
45	T T T T T . T . T T T T    
46	T T T T T . T . T T T T    
47	C C C C C . C . C C C C    
48	T T T T T . T . T T T T    
49	G G G G G . G . G G G G    
50	C C C C C . C . C C C C    
51	A A A A A . A . A A A A    
52	A A A A A . A . A A A A    
53	C C C C C . C . C C C C    
54	A A A A A . A . A A A A    
55	G G G G G . G . G G G G    
56	T T T T T . T . T T T T    
57	C C C C C . C . C C C C    
58	A A A A A . A . A A A A    
59	A A A A A . A . A A A A    
60	A A A A A . A . A A A A    
61	T T T T T . T . T T T T    
62	G G G G G . G . G G G G    
63	T T T T T . T . T T T T    
64	T T T T T . T . T T T T    
65	G G G G G . G . G G G G    
66	C C C C C . C . C C C C    
67	T T T T T . T . T T T T    
68	T T T T T . T . T T T T    
69	T T T T T . T . T T T T    
70	A A A A A . A . A A A A    
71	C C C C C . C . C C C C    
72	G G G G G . G . G G G G    
73	T T T T T . T . T T T T    
74	G G G G G . G . G G G G    
75	A A A A A . A . A A A A    
76	A A A A A . A . A A A A    
77	C C C C C . C . C C C C    
78	T T T T T . T . T T T T    
79	G G G G G . G . G G G G    
80	C C C T T . C . C C C C    
81	A A A A A . A . A A A A    
82	A A A A A . A . A A A A    
83	A A A A A . A . A A A A    
84	A A A A A . A . A A A A    
85	T T T T T . T . T T T T    
86	C C C C C . C . C C C C    
87	A A A A A . A . A A A A    
88	G G G G G . G G G G G G    
89	T T T T T . T T T T T T    
90	C C C C C . C C C C C C    
91	A A A A A . A A A A A A    
92	C C C C C . C C C C C C    
93	A A A A A . A A A A A A    
94	T T T T T . T T T T T T    
95	G G G G G . G G G G G G    
96	G G G G G . G G G G G G    
97	T T T T T . T T T T T T    
98	A A A A A . A A A A A A    
99	C C C C C . C C C C C C    
100	A A A A A . A A A A A A    
101	G G G G G . G G G G G G    
102	T T T T T . T T T T T T    
103	A A A A A . A A A A A A    
104	A A A A A . A A A G A A    
105	A A A A A . A A A A A A    
106	G G G G G . G G G G G G    
107	T T T T T . T T T T T T    
108	G G G G G . G G G G G G    
109	C C C C C . C C C C C C    
110	T T T T T . T T T T T T    
111	T T T T T . T T T T T T    
112	A A A A A . A A A A A A    
113	T T T T T . T T T T T T    
114	T T T T T . T T T T T T    
115	T T T T T . T T T T T T    
116	A A A A A . A A A A A A    
117	A A A A A . A A A A A A    
118	A A A A A . A A A A A A    
119	A A A A A . A A A A A A    
120	T T T T T . T T T T T T    
121	C C C C C . C C C C C C    
122	A A A A A . A A A A A A    
123	A A A A A . A A A A A A    
124	T T T T T . T T T T T T    
125	G G G G G . G G G G G G    
126	T T T T T . T T T T T T    
127	T T T T T . T T T T T T    
128	T T T T T . T T T T T T    
129	G G G G G . G G G G G G    
130	T T T T T . T T T T T T    
131	C C C C C . C C C C C C    
132	T T T T T . T T T T T T    
133	G G G G G . G G G G G G    
134	A A A A A . A A A A A A    
135	T T T T T . T T T T T T    
136	G G G G G . G G G G G G    
137	T T T T T . T T T T T T    
138	A A A A A . A A A A A A    
139	A A A A A . A A A A A A    
140	T T T T T . T T T T T T    
141	A A A A A . A A A A A A    
142	T T T T T . T T T T T T    
143	G G G G G . G G G G G G    
144	T T T T T . T T T T T T    
145	A A A A A . A A A A A A    
146	C C C C C . C C C C C C    
147	T T T T T . T T T T T T    
148	T T T T T . T T T T T T    
149	T T T T T . T T T T T T    
150	C C C C C . C C C C C C    
151	C C C C C . C C C C C C    
152	A A A A A . A A A A A A    
153	A A A A A . A A A A A A    
154	C C C C C . C C C C C C    
155	A A A A A . A A A A A A    
156	T T T T T . T T T T T T    
157	T T T T T . T T T T T T    
158	T T T T T . T T T T T T    
159	T T T T T . T T T T T T    
160	T T T T T . T T T T T T    
161	C C C C C . C C C C C C    
162	C C C C C . C C C C C C    
163	C C C C C . C C C C C C    
164	C C C C C . C C C C C C    
165	A A A A A . A A A A A A    
166	T T T T T . T T T T T T    
167	C C C C C . C C C C C C    
168	A A A A A . A A A A A A    
169	A A A A A . A A A A A A    
170	T T T T T . T T T T T T    
171	A A A A A . A A A A A A    
172	T T T T T . T T T T T T    
173	T T T T T . T T T T T T    
174	T T T T T . T T T T T T    
175	T T T T T . T T T T T T    
176	T T T T T . T T T T T T    
177	G G G G G . G G G G G G    
178	G G G G G . G G G G G G    
179	G G G G G . G G G G G G    
180	C C C C C . C C C C C C    
181	T T T T T . T T T T T T    
182	G G G G G . G G G G G G    
183	G G G G G . G G G G G G    
184	G G G G G . G G G G G G    
185	A A A A A . A A A A A A    
186	G G G G G . G G G G G G    
187	A A A A A . A A A A A A    
188	G G G G G . G G G G G G    
189	G G G G G . G G G G G G    
190	T T T T T . T T T T T T    
191	T T T T T . T T T T T T    
192	A A A A A . A A A A A A    
193	A A A A A . A A A A A A    
194	A A A A A . A A A A A A    
195	A A A A A . A A A A A A    
196	A A A A A . A A A A A A    
197	C C C C C . C C C C C C    
198	A A A A A . A A A A A A    
199	A A A A A . A A A A A A    
200	G G G G G . G G G G G G    
201	T T T T T . T T T T T T    
202	A A A A A . A A A A A A    
203	T T T T T . T T T T T T    
204	C C C C C . C C C C C C    
205	A A A A A . A A A A A A    
206	A A A A A . A A A A A A    
207	C C C C C . C C C C C C    
208	A A A A A . A A A A A A    
209	A A A A A . A A A A A A    
210	C C C C C . C C C C C C    
211	A A A A A . A A A A A A    
212	C C C C C . C C C C C C    
213	T T T T T . T T T T T T    
214	C C C C C . C C C C C C    
215	A A A A A . A A A A A A    
216	A A A A A . A A A A A A    
217	G G G G G . G G G G G G    
218	A A A A A . A A A A A A    
219	G G G G G . G G G G G G    
220	A A A A A . A A A A A A    
221	A A A A A . A A A A A A    
222	G G G G G . G G G G G G    
223	C C C C C . C C C C C C    
224	C C C C C . C C C C C C    
225	T T T T T . T T T T T T    
226	C C C C C . C C C C C C    
227	A A A A A . A A A A A A    
228	A A A A A . A A A A A A    
229	G G G G G . G G G G G G    
230	G G G G G . G G G G G G    
231	C C C C C . C C C C C C    
232	T T T T T . T T T T T T    
233	T T T T T . T T T T T T    
234	G G G G G . G G G G G G    
235	A A A A A . A A A A A A    
236	G G G G G . G G G G G G    
237	G G G G G . G G G G G G    
238	A A A A A . A A A A A A    
239	T T T T T . T T T T T T    
240	A A A A A . A A A A A A    
241	T T T T T . T T T T T T    
242	C C C C C . C C C C C C    
243	A A A A A . A A A A A A    
244	T T T T T . T T T T T T    
245	A A A A A . A A A A A A    
246	A A A A A . A A A A A A    
247	C C C C C . C C C C C C    
248	C C C C C . C C C C C C    
249	A A A A A . A A A A A A    
250	C C C C C . C C C C C C    
251	A A A A A . A A C A A A    
252	T T T T T . T T T T T T    
253	G G G G G . G G G G G G    
254	G G G G G . G G G G G G    
255	T T T T T . T T G T T T    
256	A A A A A . A A A A A A    
257	G G G G G . G G G G G G    
258	A A A A A . A A A A A A    
259	A A A A A . A A A A A A    
260	A A A A A A A A A A A A    
261	A A A A A A A A A A A A    
262	A A A A A A A A A A A A    
263	C C C C C C C C C C C C    
264	A A A A A A A A A A A A    
265	A A A A A A A A A A A A    
266	A A A A A A A A A A A A    
267	T T T T T T T T T T T T    
268	C C C C C C C C C C C C    
269	T T T T T T T T T T T T    
270	A A A A A A A A A A A A    
271	C C C C C C C C C C C C    
272	A A A A A A A A A A A A    
273	T T T T T T T T T T T T    
274	G G G G G G G G G G G G    
275	T T T T T T T T T T T T    
276	T T T T T T T T T T T T    
277	A A A A A A A A A A A A    
278	C C C C C C C C C C C C    
279	C C C C C C C C C C C C    
280	T T T T T T T T T T T T    
281	C C C C C C C C C C C C    
282	C C C C C C C C C C C C    
283	C C C C C C C C C C C C    
284	T T T T T T T T T T T T    
285	C C C C C C C C C C C C    
286	A A A A A A A A A A A A    
287	A A A A A A A A A A A A    
288	A A A A A A A A A A A A    
289	C C C C C C C C C C C C    
290	G G G G G G G G G G G G    
291	C C C C C C C C C C C C    
292	A A A A A A A A A A A A    
293	A A A A A A A A A A A A    
294	C C C C C C C C C C C C    
295	T T T T T T T T T T T T    
296	A A A A A A A A A A A A    
297	T T T T T T T T T T T T    
298	T T T T T T T T T T T T    
299	T T T T T T T T T T T T    
300	A A A A A A A A A A A A    
301	T T T T T T T T T T T T    
302	A A A A A A A A A A A A    
303	A A A A A A A A A A A A    
304	A A A A A A A A A A A A    
305	A A A A A A A A A A A A    
306	A A A A A A A A A A A A    
307	A A A A A A A A A A A A    
308	A A A A A A A A A A A A    
309	A A - - - A T - - T A -  6	 indel  
310	A A A A A A A A A A A A    
311	A A A A A A A A A A A A    
312	T T T T T T T T T T T T    
313	A A A A A A A A A A A A    
314	T T T T T T T T T T T T    
315	T T T T T T T T T T T T    
316	T T T T T T T T T T T T    
317	A A A A A A A A A A A A    
318	A A A A A A A A A A A A    
319	T T T T T T T T T T T T    
320	G G G G G G G G G G G G    
321	T T T T T T T T T T T T    
322	G G G G G G G G G G G G    
323	A A A A A A A A A A A A    
324	A A A A A A A A A A A A    
325	T T T T T T T T T T T T    
326	G G G G G G G G G G G G    
327	T T T T T T T T T T T T    
328	A A A A A A A A A A A A    
329	A A A A A A A A A A A A    
330	A A A A A A A A A A A A    
331	A A A A A A A A A A A A    
332	A A A A A A A A A A A A    
333	A A A A A A A A A A A A    
334	A A A A A A A A A A A A    
335	A A A A A A A A A A A A    
336	T T T T T T T T T T T T    
337	G G G G G G G G G G G G    
338	C C C C C C C C C C C C    
339	A A A A A A A A A A A A    
340	T T T T T T T T T T T T    
341	T T T T T T T T T T T T    
342	G G G G G G G G G G G G    
343	C C C C C C C C C C C C    
344	T T T T T T T T T T T T    
345	G G G G G G G G G G G G    
346	G G G G G G G G G G G G    
347	T T T T T T T T G T T T    
348	G G G G G G G G G G G G    
349	G G G G G G G G G G G G    
350	G G G G G G G G G G G G    
351	C C C C C C C C C C C C    
352	T T T T T T T T T T T T    
353	A A A A A A A A A A A A    
354	C C C C C C C C C C C C    
355	A A A A A A A A A A A A    
356	T T T T T T T T T T T T    
357	T T T T T T T T T T T T    
358	C C C C C C C C C C C C    
359	A A A A A A A A A A A A    
360	A A A A A A A A A A A A    
361	A A A A A A A A A A A A    
362	T T T T T T T T T T T T    
363	T T T T T T T T T T T T    
364	G G G G G G G G G G G G    
365	T T T T T T T T T T T T    
366	G G G G G G G G G G G G    
367	G G G G G G G G G G G G    
368	G G G G G G G G G G G G    
369	G G G G G G G G G G G G    
370	A A A A A A A A A A A A    
371	A A A A A A A A A A A A    
372	G G G G G G G G G G G G    
373	T T T T T T T T T T T T    
374	G G G G G G G G G G G G    
375	G G G G G G G G G G G G    
376	A A A A A A A A A A A A    
377	T T T T T T T T T T T T    
378	T T T T T T T T T T T T    
379	A A A A A A A A A A A A    
380	T T T T T T T T T T T T    
381	G G G G G G G G G G G G    
382	G G G G G G G G G G G G    
383	C C C C C C C C C C C C    
384	T T T T T T T T T T T T    
385	C C C C C C C C C C C C    
386	T T T T T T T T T T T T    
387	C C C C C C C C C C C C    
388	C C C C C C C C C C C C    
389	A A A A A A A A A A A A    
390	G G G G G G G G G G G G    
391	T T T T T T T T T T T T    
392	T T T T T T T T T T T T    
393	G G G G G G G G G G G G    
394	A A A A A A A A A A A A    
395	C C C C C C C C C C C C    
396	T T T T T T T T T T T T    
397	T T T T T T T T T T T T    
398	T T T T T T T T T T T T    
399	A A A A A A A A A A A A    
400	T T T T T T T T T T T T    
401	A A A A A A A A A A A A    
402	A A A A A A A A A A A A    
403	A A A A A A A A A A A A    
404	A A A A A A A A A A A A    
405	G G G G G G G G G G G G    
406	G G G G G G G G G G G G    
407	T T T T T T T T G T T T    
408	C C C C C C C C C C C C    
409	T T T T T T T T T T T T    
410	T T T T T T T T T T T T    
411	A A A A A A A A A A A A    
412	C C C C C C C C C C C C    
413	A A A A A A A A A A A A    
414	A A A A A A A A A A A A    
415	A A A A A A A A A A A A    
416	A A A A A A A A A A A A    
417	A A A A A A A A A A A A    
418	G G G G G G G G G G G G    
419	C C C C C C C C C C C C    
420	T T T T T T T T T T T T    
421	A A A A A A A A A A A A    
422	A A A A A A A A A A A A    
423	A A A A A A A A A A A A    
424	A A A A A A A A A A A A    
425	A A A A A A A A A A A A    
426	A A A A A A A A A A A A    
427	A A A A A A A A A A A A    
428	A A A A A A A A A A A A    
429	C C - C C C C C A C C C    
430	A A A A A A A A A A A A    
431	A A A A A A A A A A A A    
432	- - C - - - C - C C - -  4	 indel  
433	- - T - - - A - T A - -    
434	- - A - - - A - A A - -  4	 indel  
435	A A A A A A A A A A A A    
436	T T T T T T T T T T T T    
437	A A A A A A A A A A A A    
438	T T T T T T T T T T T T    
439	T T T T T T T T T T T T    
440	T T T T T T T T T T T T    
441	A A A A A A A A A A A A    
442	A A A A A A A A A A A A    
443	A A A A A A A A A A A A    
444	A A A A A A A A A A A A    
445	A A A A A A A A A A A A    
446	G G G G G G G G G G G G    
447	C C C C C C C C C C C C    
448	T T T T T T T T T T T T    
449	A A A A A A A A A A A A    
450	G G G G G G G G G G G G    
451	G G G G G G G G G G G G    
452	C C C C C C C C C C C C    
453	A A A A A A A A A A A A    
454	T T T T T T T T T T T T    
455	A A A A A A A A A A A A    
456	G G G G G G G G G G G G    
457	C C C C C C C C C C C C    
458	T T T T T T T T T T T T    
459	T T T T T T T T T T T T    
460	G G G G G G G G G G G G    
461	T T T T T T T T T T T T    
462	A A A A A A A A A A A A    
463	T T T T T T T T T T T T    
464	T T T T T T T T T T T T    
465	C C C C C C C C C C C C    
466	A A A A A A A A A A A A    
467	T T T T T T T T T T T T    
468	T T T T T T T T T T T T    
469	C C C C C C C C C C C C    
470	T T T T T T T T T T T T    
471	C C C C C C C C C C C C    
472	T T T T T T T T T T T T    
473	C C C C C C C C C C C C    
474	C C C C C C C C C C C C    
475	A A A A A A A A A A A A    
476	A A A A A A A A A A A A    
477	A A A A A A A A A A A A    
478	T T T T T T T T T T T T    
479	A A A A A A A A A A A A    
480	A A A A A A A A A A A A    
481	A A A A A A A A A A A A    
482	A A A A A A A A A A A A    
483	. A A A A A A A A A A A    
484	. G G G G G G G G G G G    
485	. G G G G G G G G G G G    
486	. C C C C C C C C C C C    
487	. A A A A A A A A A A A    
488	. G G G G G G G G G G G    
489	. T T T T T T T T T T T    
490	. C C C C C C C C C C C    
491	. C C C C C C C C C C C    
492	. A A A A A A A A A A A    
493	. A A A A A A A A A A A    
494	. A A A A A A A A A A A    
495	. A A A A A A A A A A A    
496	. C C C C C C C C C C C    
497	. T T T T T T T T T T T    
498	. A A A A A A A A A A A    
499	. C C C C C C C C C C C    
500	. T T T T T T T T T T T    
501	. G G G G G G G G G G G    
502	. C C C C C C C C C C C    
503	. A A A A A A A A A A A    
504	. T T T T T T T T T T T    
505	. T T T T T T T T T T T    
506	. G G G G G G G G G G G    
507	. G G G G G G G G G G G    
508	. G G G G G G G G G G G    
509	. T T T T T T T T T T T    
510	. A A A A A A A A A A A    
511	. A A A A A A A A A A A    
512	. A A A A A A A A A A A    
513	. A A A A A A A A A A A    
514	. G G G G G G G G G G G    
515	. T T T T T T T T T T T    
516	. A A A A A A A A A A A    
517	. G G G G G G G G G G G    
518	. A A A A A A A A A A A    
519	. A A A A A A A A A A A    
520	. A A A A A A A A A A A    
521	. T T T T T T T T T T T    
522	. G G G G G G G G G G G    
523	. A A A A A A A A A A A    
524	. T T T T T T T T T T T    
525	. T T T T T T T T T T T    
526	. A A A A A A A A A A A    
527	. T T T T T T T T T T T    
528	. G G G G G G G G G G G    
529	. A A A A A A A A A A A    
530	. C C C C C C C C C C C    
531	. G G G G G G G G G G G    
532	. A A A A A A A A A A A    
533	. A A A A A A A A A A A    
534	. G G G G G G G G G G G    
535	. G G G G G G G G G G G    
536	. C C C C C C C C C C C    
537	. A A A A A A A A A A A    
538	. A A A A A A A A A A A    
539	. C C C C C C C C C C C    
540	. T T T T T T T T T T T    
541	. C C C C C C C C C C C    
542	. A A A A A A A A A A A    
543	. T T T T T T T T T T T    
544	. A A A A A A A A A A A    
545	. C C C C C C C C C C C    
546	. C C C C C C C C C C C    
547	. T T T T T T T T T T T    
548	. T T T T T T T T T T T    
549	. A A A A A A A A A A A    
550	. A A A A A A A A A A A    
551	. T T T T T T T T T T T    
552	. T T T T T T T T T T T    
553	. A A A A A A A A A A A    
554	. T T T T T T T T T T T    
555	. A A A A A A A A A A A    
556	. T T T T T T T T T T T    
557	. C C C C C C C C C C C    
558	. T T T T T T T T T T T    
559	. A A A A A A A A A A A    
560	. A A A A A A A A A A A    
561	. A A A A A A A A A A A    
562	. G G G G G G G G G G G    
563	. A A A A A A A A A A A    
564	. G G G G G G G G G G G    
565	. T T T T T T T T T T T    
566	. A A A A A A A A A A A    
567	. C C C C C C C C C C C    
568	. T T T T T T T T T T T    
569	. T T T T T T T T T T T    
570	. C C C C C C C C C C C    
571	. C C C C C C C C C C C    
572	. C C C C C C C C C C C    
573	. A A A A A A A A A A A    
574	. A A A A A A A A A A A    
575	. G G G G G G G G G G G    
576	. T T T T T T T T T T T    
577	. C C C C C C C C C C C    
578	. T T T T T T T T T T T    
579	. T T T T T T T T T T T    
580	. T T T T T T T T T T T    
581	. G G G G G G G G G G G    
582	. T T T T T T T T T T T    
583	. G G G G G G G G G G G    
584	. C C C C C C C C C C C    
585	. A A A A A A A A A A A    
586	. T T T T T T T T T T T    
587	. T T T T T T T T T T T    
588	. T T T T T T T T T T T    
589	. C C C C C C C C C C C    
590	. T T T T T T T T T T T    
591	. G G G G G G G G G G G    
592	. A A A A A A A A A A A    
593	. G G G G G G G G G G G    
594	. A A A A A A A A A A A    
595	. A A A A A A A A A A A    
596	. T T T T T T T T T T T    
597	. C C C C C C C C C C C    
598	. C C C C C C C C C C C    
599	. C C C C C C C C C C C    
600	. A A A A A A A A A A A    
601	. A A A A A A A A A A A    
602	. C C C C C C C C C C C    
603	. A A A A A A A A A A A    
604	. A A A A A A A A A A A    
605	. A A A A A A A A A A A    
606	. A A A A A A A A A A A    
607	. C C C C C C C C C C C    
608	. G G G G G G G G G G G    
609	. T T T T T T T T T T T    
610	. A A A A A A A A A A A    
611	. G G G G G G G G G G G    
612	. A A A A A A A A A A A    
613	. G G G G G G G A G G G    
614	. A A A A A A A A A A A    
615	. A A A A A A A A A A A    
616	. A A A A A A A A A A A    
617	. A A A A A A A A A A A    
618	. A A A A A A A A A A A    
619	. A C C C C C C C C C C    
620	. . A A A A A A A A A A    
621	. . T T T T T T T T T T    
622	. . T T T T T T T T T T    
623	. . A A A A A A A A A A    
624	. . G G G G G G A G G G    
625	. . A A A A A A A A A A    
626	. . T T T T T T T T T T    
627	. . A A A A A A A A A A    
628	. . C C C C C C C C C C    
629	. . T T T T T T T T T T    
630	. . T T T T T T T T T T    
631	. . T T T T T T T T T T    
632	. . T T T T T T T T T T    
633	. . T T T T T T T T T T    
634	. . T T T T T T T T T T    
635	. . T T T T A T T T T T    
636	. . T A A A A A T A A A    
637	. . . A A A A A A A A A    
638	. . . A A A A A A A A A    
639	. . . A A A - A A A A A    
640	. . . G G G G G G G G G    
641	. . . T T T T T T T T T    
642	. . . A A A A A A A A A    
643	. . . G G G G G G G G G    
644	. . . A A C A A A A A A    
645	. . . A A . A A A A A A    
646	. . . A A . A A A A A A    
647	. . . C C . C C C C C C    
648	. . . T T . T T T T T T    
649	. . . G G . G G G G G G    
650	. . . A A . A A A A A A    
651	. . . A A . A A A A A A    
652	. . . T T . T T T T T T    
653	. . . T T . T T T T T T    
654	. . . G G . G G G G G G    
655	. . . C C . A A A A A A    
656	. . . . . . A A A A A A    
657	. . . . . . T T T T T T    
658	. . . . . . A A A A A A    
659	. . . . . . T T T T T T    
660	. . . . . . G G G G G G    
661	. . . . . . T T T T T T    
662	. . . . . . A A A A A A    
663	. . . . . . T T T T T T    
664	. . . . . . T T T T T T    
665	. . . . . . T T T T T T    
666	. . . . . . T T T T T T    
667	. . . . . . G G G G G G    
668	. . . . . . A A A A A A    
669	. . . . . . A A A A A A    
670	. . . . . . A A A A A A    
671	. . . . . . A A A A A A    
672	. . . . . . C C C C C C    
673	. . . . . . C C C C C C    
674	. . . . . . C C C C C C    
675	. . . . . . T T T T T T    
676	. . . . . . A A . A A A    
677	. . . . . . A A . A A A    
678	. . . . . . A A . A A A    
679	. . . . . . G G . G G G    
680	. . . . . . A A . A A A    
681	. . . . . . C C . C C C    
682	. . . . . . C C . C C C    
683	. . . . . . G G . G G G    
684	. . . . . . C C . C C C    
685	. . . . . . C C . C C C    
686	. . . . . . T T . T T T    
687	. . . . . . T T . T T T    
688	. . . . . . A A . A A A    
689	. . . . . . C C . C C C    
690	. . . . . . T T . T T T    
691	. . . . . . T T . T T T    
692	. . . . . . A A . A A A    
693	. . . . . . G G . G G G    
694	. . . . . . C C . C C C    
695	. . . . . . T T . T T T    
696	. . . . . . T T . T T T    
697	. . . . . . A A . A A A    
698	. . . . . . A A . A A A    
699	. . . . . . T T . T T T    
700	. . . . . . G G . G G G    
701	. . . . . . C C . C C C    
702	. . . . . . . T . T T T    
703	. . . . . . . T . T T T    
704	. . . . . . . T . T T T    
705	. . . . . . . T . T T T    
706	. . . . . . . G . G G G    
707	. . . . . . . T . T T T    
708	. . . . . . . T . T T T    
709	. . . . . . . C . C C C    
710	. . . . . . . A . A A A    
711	. . . . . . . G . G G G    
712	. . . . . . . A . A A A    
713	. . . . . . . T . T T T    
714	. . . . . . . T . T T T    
715	. . . . . . . C . C C C    
716	. . . . . . . T . T T T    
717	. . . . . . . T . T T T    
718	. . . . . . . T . G G G    
719	. . . . . . . . . A A A    
720	. . . . . . . . . T T T    
721	. . . . . . . . . A A A    
722	. . . . . . . . . G G G    
723	. . . . . . . . . C C C    
724	. . . . . . . . . A A A    
725	. . . . . . . . . A A A    
726	. . . . . . . . . G G G    
727	. . . . . . . . . . G G    
728	. . . . . . . . . . T T    
729	. . . . . . . . . . A A    
730	. . . . . . . . . . G G    
731	. . . . . . . . . . T T    
732	. . . . . . . . . . T T    
733	. . . . . . . . . . T T    
734	. . . . . . . . . . C C    
735	. . . . . . . . . . C A    
736	. . . . . . . . . . . C    
737	. . . . . . . . . . . A    
738	. . . . . . . . . . . A    
739	. . . . . . . . . . . A    
740	. . . . . . . . . . . C    
741	. . . . . . . . . . . A    
742	. . . . . . . . . . . A    
743	. . . . . . . . . . . A    
744	. . . . . . . . . . . G    
745	. . . . . . . . . . . A    
746	. . . . . . . . . . . A    
747	. . . . . . . . . . . A    
748	. . . . . . . . . . . A    
749	. . . . . . . . . . . C    
750	. . . . . . . . . . . A    
751	. . . . . . . . . . . G    
752	. . . . . . . . . . . C    
753	. . . . . . . . . . . T    
754	. . . . . . . . . . . G    
755	. . . . . . . . . . . C    
756	. . . . . . . . . . . A    
757	. . . . . . . . . . . T    
758	. . . . . . . . . . . A    
759	. . . . . . . . . . . C    
760	. . . . . . . . . . . A    
761	. . . . . . . . . . . C    
762	. . . . . . . . . . . A    
763	. . . . . . . . . . . T    
764	. . . . . . . . . . . T    
765	. . . . . . . . . . . C    
766	. . . . . . . . . . . A    
767	. . . . . . . . . . . A    
768	. . . . . . . . . . . G    
769	. . . . . . . . . . . T    
770	. . . . . . . . . . . A    
771	. . . . . . . . . . . A    
772	. . . . . . . . . . . G    
773	. . . . . . . . . . . T    
774	. . . . . . . . . . . C    
775	. . . . . . . . . . . T    
776	. . . . . . . . . . . T    
777	. . . . . . . . . . . A    
778	. . . . . . . . . . . C    
779	. . . . . . . . . . . T    
780	. . . . . . . . . . . A    
781	. . . . . . . . . . . T
```
